# Supplementary figures and images for: Relationship of maternal cytomegalovirus-specific antibody responses and viral load to vertical transmission risk following primary maternal infection in a rhesus macaque model
Source: PLoS Pathog. 2023 Oct 23;19(10):e1011378. doi: 10.1371/journal.ppat.1011378 (PMC10621917; doi:10.1371/journal.ppat.1011378)

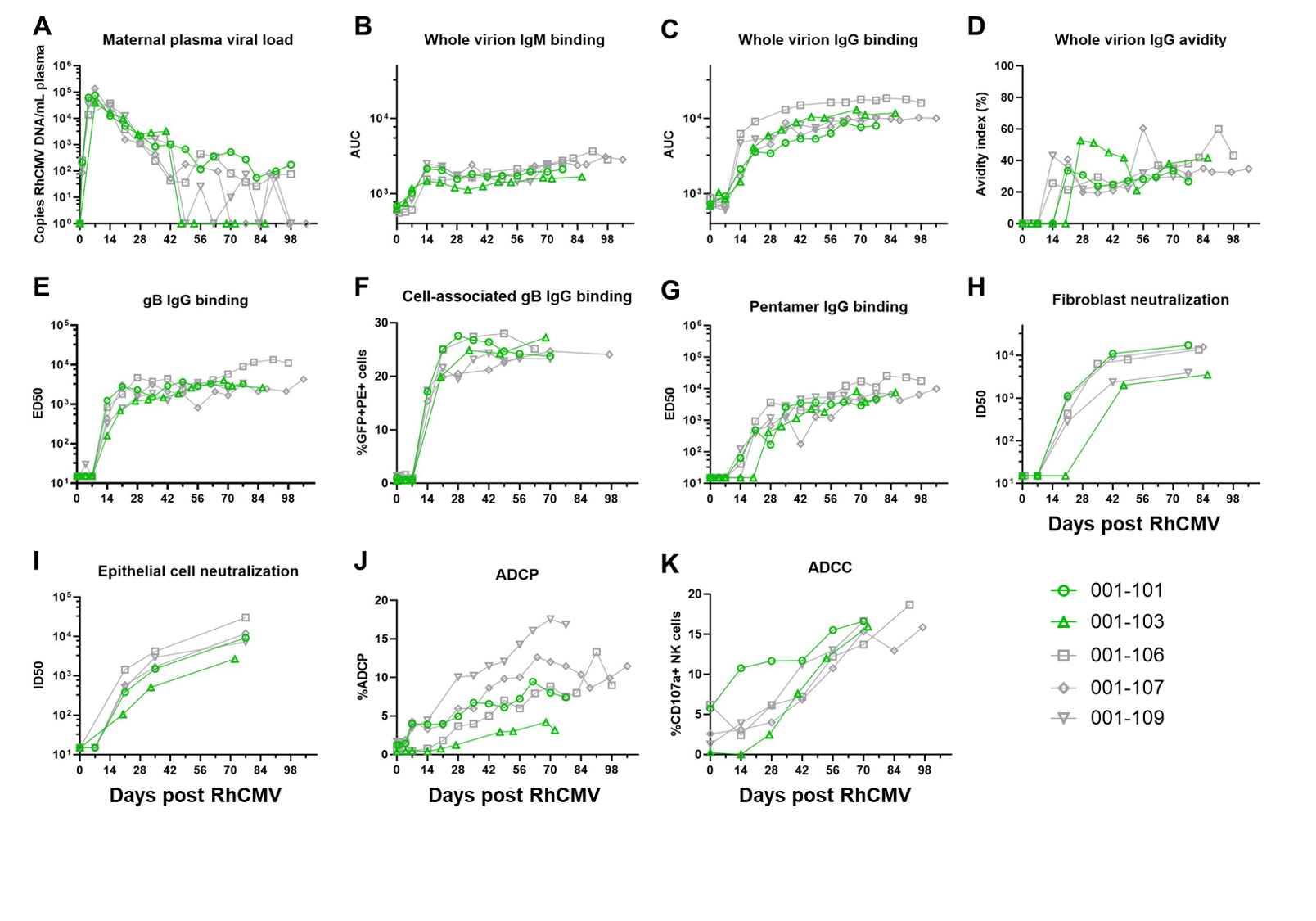

Supplement: S1 Fig — (A) Maternal plasma VL measured by qPCR for the UL55 or IE genes; (B) IgM and (C) IgG binding to whole UCD52 RhCMV virions; (D) avidity of IgG binding against whole UCD52 virions; IgG binding to (E) PC, (F) soluble gB ectodomain by ELISA, and (G) cell-associated gB via transfected cell binding assay; antibody mediated RhCMV neutralization on (H) fibroblasts and (I) epithelial cells; (J) ADCP; and (K) ADCC. (TIF) [file ppat.1011378.s001.tif]

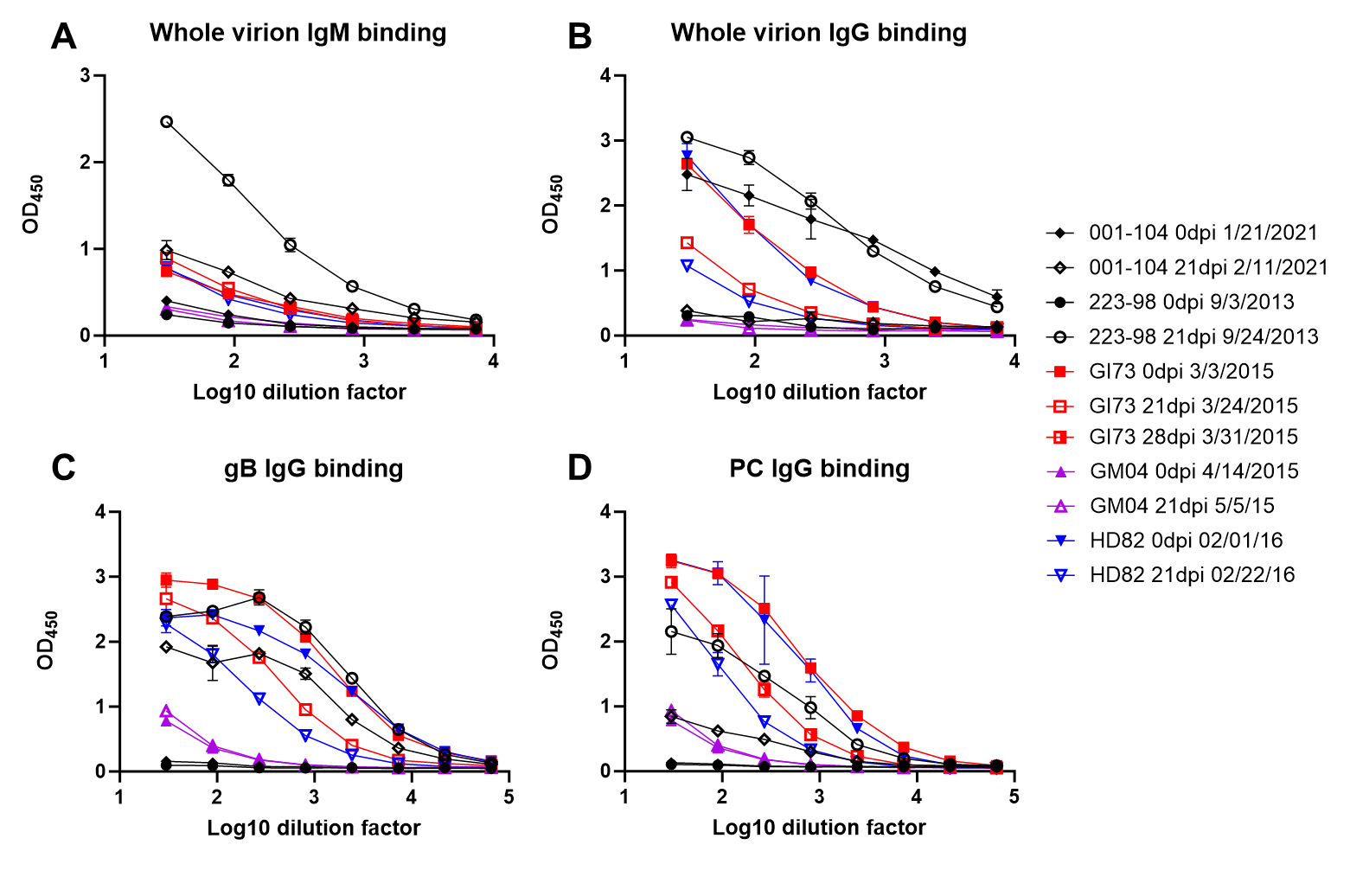

Supplement: S2 Fig — The optical density at 450 nm (OD450) are plotted versus log dilution factor for (A) whole virion IgM binding, (B) whole virion IgG binding, (C) gB IgG binding, and (D) PC IgG binding. One animal from each treatment group is represented, showing the samples taken at the time of infection and 21- or 28-days post infection. (TIF) [file ppat.1011378.s002.tif]
