# Supplementary material for: Relationship of maternal cytomegalovirus-specific antibody responses and viral load to vertical transmission risk following primary maternal infection in a rhesus macaque model
Source: PLoS Pathog. 2023 Oct 23;19(10):e1011378. doi: 10.1371/journal.ppat.1011378 (PMC10621917; doi:10.1371/journal.ppat.1011378)
Supplement: Attachment — Submitted filename: 20220913 response to reviewers_v3.docx [file ppat.1011378.s003.docx]

**Point-by-point response to reviewer comments**

The authors would like to thank the reviewers for their comments and thorough review of this manuscript. We are grateful for the valuable feedback and appreciate the opportunity to submit this revision. We have addressed the reviewers’ comments as below.

**Part I - Summary**
Please use this section to discuss strengths/weaknesses of study, novelty/significance, general execution and scholarship.

Reviewer #1: This study by Otero et al. is highly significant as it provides the first in vivo model that appears to closely mimic human mother-to-fetus vertical transmission following a primary HCMV infection. Primary infection of pregnant persons results in an ~40% transmission rate. However, identifying immunological and/or virological factors that provide protection against vertical transmission following a primary HCMV infection is a major gap in the field largely due to the asymptomatic/mild nature of infection in healthy individuals and the lack of a biologically comparable in vivo model. The Permar group originally developed a non-human primate (NHP) model of vertical transmission by depleting maternal CD4 T cells prior of RhCMV challenge, which resulted in a 100% mother-to-fetus transmission rate. This current study adds to those initial findings by increasing the cohort size of fully immunocompetent seronegative rhesus dams to assess RhCMV transmission in healthy mothers. Remarkably, similar to HCMV vertical transmission rates, the investigators found an ~40% RhCMV transmission rate in seronegative rhesus dams, suggesting this model could identify key immunological and/or virological determinates of congenital CMV transmission in healthy pregnant persons. Using this model, the investigators perform standard virological and immunological assays to attempt to identify factors associated with promoting or opposing RhCMV vertical transmission. They found neither levels of maternal viremia nor humoral responses were associated with congenital CMV infection following a primary infection of healthy dams, which was unknown prior to development of this model. From a research standpoint, the individual experimental assays are straightforward and appropriate. However, the presentation of some of the data are difficult to follow at times, particularly the results combining both healthy and CD4-depleted dams. Nonetheless, this is a highly significant study describing a novel NHP vertical transmission model that will provide the means to address previously unanswerable questions about mechanisms/correlates of protection in healthy pregnancy persons following a primary CMV infection.

- *Thank you for this summary and positive comments. We have made an effort to make the results easier to follow in this revision by clarifying which group is being discussed in each sentence.*

Reviewer #2: This manuscript describes use of the rhesus macaque model to determine possible humoral correlates of protection against congenital transmission of rhesus cytomegalovirus (RhCMV), an important model for human cytomegalovirus congenital disease. Twelve RhCMV-negative dams were infected IV with a cocktail of two RhCMV strains. Blood and amniotic fluid (AF) were collected weekly and analyzed for viral load. Plasma was assessed for RhCMV-specific antibodies, neutralizing antibodies, and antibodies mediating RhCMV-specific ADCP and ADCC. Five dams had PCR-positive signals for RhCMV DNA in AF while seven were negative out to 20 weeks post challenge. There were no apparent associations between maternal viral load or the above measured humoral responses with fetal transmission.

The authors then added to these studies data from 15 additional experimentally-infected RhCMV-negative dams from previously published studies that were in some cases CD4-depleted and infused prior to infection with two RhCMV hyperimmuneglobulin (HIG) preparations. In some cases data were incorporated from the previously published studies, while some assays were conducted contemporaneously using archival samples. Incorporation of these additional animals generated inconsistencies that could confound interpretation, particularly as animals were infected with different viral cocktails consisting of different strains and two different HIG infusion protocols were used.

The resulting “meta” analyses identified high maternal viral load and decreased/delayed development of gB- and PC-specific binding antibodies as associated with transmission, but these correlations were largely driven by the CD4-depleted group, which considerably confounded interpretation. However, the data overall suggested that antibodies can provide protection against fetal infection, but in the context of primary infection they may not be present early enough to prevent transmission.

In general the manuscript is well organized and clearly written. Materials and Methods are a bit sloppy; detailed suggestions for improvement are provided among the “specific comments”. There are a few broad topics, presented below, that the authors may wish to consider elaborating on in the Discussion.

There is evidence that in humans delayed maternal humoral responses after primary infection are associated with transmission (PMID: 28008685). There is also evidence that HIG can reduce transmission if given early, at high dose, and more frequently than standard regimens (PMID: 29947159). It is also surprising that the authors did not choose to measure antibody avidity, which has been hypothesized to facilitate virion transport across the placenta. In humans high avidity antibodies can take two to six months to evolve and timing can be variable. The rhesus studies spanned about three months and so it is possible that the late transmission events may be associated with lower antibody avidity.

Congenital transmission is a highly complex and dynamic process that evolves dramatically during the course of pregnancy. The rhesus studies described raise important questions regarding time of transmission relative to both infection and conception. The authors allude to fetal transmission occurring rapidly after IV challenge in the rhesus model, but their data do not seem to support this, as all but one of the AF-positive dams had their first AF-positive result four weeks or more after challenge. Can the fetus be infected early but virus not be detected in the AF for several more weeks? In humans is there evidence that transmission occurs shortly after initial primary maternal infection, or is it a more protracted process?

It is also important to consider that the mechanisms of transplacental transmission, and consequently immune protection, may be different in first vs third trimester. In humans CMV transmission to the fetus is more efficient in third trimester than in first, perhaps because the integrity of the placenta as a maternal-fetal blood barrier begins to decline late in pregnancy. Thus transmissions that occur late in pregnancy may simply be due to mechanical leakage, whereas those early in pregnancy may require more active mechanisms such as progressive spreading of virus infections across placental tissues or, as has been proposed, active transport of virions via low avidity non-neutralizing antibodies. In any case, and especially as third trimester transmissions are also associated with less severe disease, it may make sense to analyze data from early fetal infections separately from later infections. For example, 0-3 week AOC data could be correlated with fetal infections that occurred up to 28 days post infection, while 3+ week AOC data could be correlated with those that occurred late (70-84 days).

While cumulatively the results reported are largely negative and in some instances are insufficient to support robust conclusions, the importance of identifying correlates of immune protection against congenital CMV disease, the paucity of data from this valuable animal model, and the thoroughness of the humoral analyses presented render this work of high significance to the field.

- *Thank you for such a thorough summary and positive comments regarding the significance of the work. We appreciate the constructive criticism and have made an effort to clean up the Methods following the reviewer’s specific recommendations. We additionally conducted avidity analysis (Figs. 2, 4, 6, and 7) as the reviewer recommended, as we agreed that this would be an important addition to this study. While we agree that the timing of fetal infection is an important factor to consider, we unfortunately do not have statistical power to perform further subset analysis regarding early versus late transmission as this would be n = 3 versus n = 2, respectively, within the recent 12-dam cohort. We have instead added visualization of the kinetics of each response with “late transmitters” colored in green (Fig S1).*

Reviewer #3: Otero et al evaluate cytomegalovirus “transmission” in a limited number of rhesus macaques in order to define correlates of congenital CMV (cCMV) infection. cCMV is a highly significant disease in human populations, and insights into how risk of transmission might be reduced are a clear and compelling clinical need. To the extent possible by abstracting from an animal model, this study demonstrates that neither maternal plasma viremia nor humoral immune responses relate to detection of CMV in amniotic fluid. The study is small (12 animals) and relies on several simplifications (equating amniotic fluid viral detection with fetal transmission) and abstractions between both host (NHP vs humans) and pathogen (rhCMV vs hCMV).

Beyond grammatical tidying, the manuscript would be improved by a more serious discussion of the limitations of the study. Some suggests are listed below.

- *Thank you for the summary and constructive comments. We have worked to implement the reviewer’s suggestions, including expansion of a discussion of the limitations of the study in the Discussion.*

**Part II – Major Issues: Key Experiments Required for Acceptance**
Please use this section to detail the key new experiments or modifications of existing experiments that should be absolutely required to validate study conclusions.

Generally, there should be no more than 3 such required experiments or major modifications for a "Major Revision" recommendation. If more than 3 experiments are necessary to validate the study conclusions, then you are encouraged to recommend "Reject".

Reviewer #1:

1. Figure 1C shows the timing of detection of vertical transmission in the amniotic fluid (AF). It would also be helpful to the reader to show RhCMV DNA copies and duration of viral load in the AF of the 5 dams that exhibited vertical transmission in order to get a full sense of the magnitude of transmission during pregnancy.

- *This data will be incorporated into a separate manuscript led by Dr. Kaur which describes the full virologic and pathologic outcomes of the dams and fetuses in much more detail, which we hope will be pre-printed prior to the publication of this manuscript so we can cite this work. For the purposes of this immune correlate study, we utilized RhCMV detection in amniotic fluid as a binary variable to represent transmission status.*

2. In figure 2, the area under the curve (AUC) presented for the full virion serology are difficult to interpret because of the use of AUC rather than EC50 or endpoint titers, especially since EC50 are used for the other panels. Can these data be presented as endpoint titers or EC50 to improve clarity. It’s unclear as to how the AUC results represent the absolute fold change in antibody levels (i.e., does a 10-fold change in AUC equal a 10-fold antibody abundance).

- *We agree that ED50s are the optimal way to report antibody titers, but we opted to utilize AUC for whole virion ELISAs because samples rarely yielded a sufficient sigmoidal curve for calculating ED50s. We additionally preferred AUC over endpoint titer because endpoint titer is a discrete value, and for the purposes of the analysis we performed, a continuous variable was more appropriate. In order to provide more details on the response to allow for the reader’s full interpretation, the OD curves of the IgG binding to whole virions are included in supplemental data (Fig S2).*

3. Maternal IgG transfer across the placenta provides protection for the fetus from infection. Reduced levels of maternal IgG in the fetal circulation could lead to vertical transmission. What are the levels of maternal IgG in the umbilical cord blood (fetal circulation)?

- *We agree that this is an important analysis, but we feel it is beyond the scope of this work as the primary goal of this study was to identify maternal immune responses that associate with protection to inform immune targets for vaccine development. This information will also be included in the separate paper being put together on the fetal outcomes led by Dr. Kaur.*

4. Given that one of the main functions of the placenta is to act as a barrier protecting the fetus from infection, placental pathology (gross and/or microscopic) should be provided comparing AF-positive and AF-negative dams.

- *This information outside of scope for the maternal humoral immune correlate analysis that we performed and will also be included with a second manuscript which describes the animal model and placental/fetal outcomes in more detail.*

5. In figure 6, the investigators combine data from a previous study using CD4 T cell depleted dams that were treat with or without passive RhCMV-specific IgG antibodies. However, combining data from immunocompetent dams with those from immunocompromised dams somewhat muddles the results. Plotting AUC data only from the immunocompetent dams separating those that exhibited vertical transmission versus those that did not would be far more informative. Immunocompromised dams that did or did not receive passive IgG infusions could be plotted in a separate figure.

- *As recommended by this reviewer, the AUC values for immunocompetent and CD4-depleted dams are now represented in separate figures (Fig 6 and 7).*

Reviewer #2: None required, but the authors should consider looking at avidity.

- *Thank you for this suggestion. We measured IgG avidity and included it in our analysis.*

Reviewer #3:

Comment on the on the challenge? Differences between the viruses? Can they be read out independently?

- *All animals received the RhCMV primary isolate UCD52 in combination with one or two other strains (primary isolate UCD59, a recombinant full length RhCMV strain based on strain 68.1, and the fibroblast adapted 180.92), except for the one animal that only received the fibroblast-adapted RhCMV strain180.92. Immunologic analysis would be difficult to differentiate between the four strains used in these studies, as CMV strains do not exist as serotypes, but prior work in the historical cohort (Bialas et al…) and ongoing work in the new 12 dam cohort has utilized virus sequencing to estimate the relative proportions of each strain via sequencing analysis. This extensive sequencing analysis for the new cohort is within the scope of the other manuscript being developed which describes the virologic outcomes of model in much more detail.*

Tabulate and comment on effect size in addition to statistical significance.

- *Effect size was added to Table 1 for each pairwise comparison.*

Comment on power given small study size. What magnitude of effect could have been reliably detected with 12 animals? Especially given the variance observed in viral loads and humoral responses?

- *Thank you for the comment. Post-hoc power analyses are generally non-informative after the analyses have been performed. As this is the case, the probability of us rejecting the null hypothesis is 0 or 1 and thus the post-hoc power will be fundamentally flawed (Lyderson 2014). We also direct the reviewer to articles by Gelman (2019) and Hoenig & Heisey (2001). These articles discuss how post-hoc power is dependent on the observed data which may give unrealistic expectations of power. Based on these discussions, we believe that performing post-hoc power calculations will not add to the paper. We have however added to the discussion, further acknowledging of the small sample size.*

*Lydersen S. Statistical review: frequently given comments. Ann Rheum Dis. 2015; 74(2): 323-5.*

*Hoenig JM, Heisey DM. The Abuse of Power. The American Statistician. 2001; 55(1): 19-24.*

*Gelman, A. Don’t Calculate Post-hoc Power Using Observed Estimate of Effect Size. Ann Surg. 2019; 269(1): e9-e10.*

Describe limitations of modeling CMV – different virus, different route and kinetics of infection, AF, differences in Ab biology between humans and rhesus, AF virus status as a proxy for fetal infection, etc.

- *The challenges in modeling CMV were introduced in the Introduction when the model system was justified (Page 4), but a more thorough discussion of the limitations of the rhesus model has been added to the discussion (Page 13).*

**Part III – Minor Issues: Editorial and Data Presentation Modifications**
Please use this section for editorial suggestions as well as relatively minor modifications of existing data that would enhance clarity.

Reviewer #1:

1. In figure 6, the investigators combine data from a previous study using CD4 T cell depleted dams that were treat with or without passive RhCMV-specific IgG antibodies. However, combining data from immunocompetent dams with those from immunocompromised dams somewhat muddles the results. Plotting AUC data only from the immunocompetent dams separating those that exhibited vertical transmission versus those that did not would be far more informative. Immunocompromised dams that did or did not receive passive IgG infusions could be plotted in a separate figure.

- *AUC values for immunocompetent and CD4-depleted dams are now represented in separate figures (Fig 6 and 7).*

Reviewer #2: Specific comments:
1. 78-79. NHP CMVs include CMVs that infect Great Apes. *The reviewer is correct, this has been corrected.*
2. 83. “fraction of the time” is vague and also suggests a short time period rather than low incidence; suggest “rarely” or similar. *We agree, this has been changed to “in a fraction of immunocompetent dams“.*
3. 141. Should be “Figure 3A, B”. *Thank you for catching this typo. Corrected.*
4. 144. “never reached”. *This has been corrected.*
5. 148-149. Unclear what is meant by “dose-optimized”. *In this historical study, the IgG infusion dosing for a second group of dams was informed by PK analysis on the initial group of HIG-infused dams, with the second group of dams receiving two IgG infusions to maintain a high level of antibody through the first week post infection. This language was removed to avoid confusion.*
6. 152-154. It would be helpful to briefly describe the different virus strains used in these studies. Are there four different strains, or is FL a BAC clone of 180.92? Do they have relevant genetic features or cell tropisms (e.g., PC mutations)? *Additional details on the challenge virus was added in the results section (Page 6-7).*
7. 164-176. This paragraph was very hard to follow. In particular, it is unclear what group of animals are referred to in lines 168-169, or what exactly is the “combined group”. Also, please clarify “higher magnitude” - compared to what group (presumably AF-negative, but it is not stated)? *This section was edited for clarity as recommended.*
8. 195. Do you mean Fig. 7 here? *Yes, this has been corrected.*
9. 197. “suggested”. *Corrected.*
10. 230-233. The importance of early HIG treatment is further supported by work from Klaus Hamprecht (PMID: 29947159). *We have included the additional citation and updated the language of this statement to ensure accuracy with the additional evidence.*
11. 238-240. The AF data presented seem inconsistent with this statement as four of the five AF-positives were first detected >28 days post infection and two were not detected until >70 days. Are there other data to suggest that transmission in this model occurs “shortly following infection”?

*Yes, this was inclusive of historical results, which we realize from this comment was not explicitly shown. We have added the survival curves of all groups to Figure 5 to demonstrate this.*
12. 260-262. “high titer” compared to what? *Clarified relative to other dam groups.*
13. 320-331. Please provide species, source or reference, and catalog numbers (where available) for all cell lines. Include 1RFs (line 346) in this section. *Information on 1RFs has been moved to this section and additional information added for the other cell lines included as indicated by the reviewer.*
14. 335. Please clarify “reduced-FBS”. *This has been clarified.*
15. 342-344. Please provide details or reference for virus storage and titration methods. *Added.*
16. 346-348. Please provide references or brief details of BAC construction for FL-RhCMV, including strain. *Reference added.*
17. 405-406. Please provide references or brief details about the plasmids used. *Additional information about the GFP plasmid was added.*
18. 425. Were these data background-corrected with negative plasma? *This was clarified to explain that the gating set a cutoff based on the no antibody control wells.* *Seronegative samples were included in every run and the background level, which is quite low, can be seen from the time 0 samples in Figure 3C.*
19. 445. Were these data also background-corrected with negative plasma? *The background was subtracted first from no antibody control wells for both mock and infected conditions and then the mock condition was subtracted from the infected for each respective sample. Seronegative samples were included as negative controls in every run and the background level, which can be variable, is presented as time 0 samples in Figure 3D.*
20. 598-599. This statement is not accurate as one animal was infected only with 180.92 and 12 were infected with UCD52 and FL. *Virus dosing information was added to the Figure 5 to improve clarity.*
21. 608. This figure does not appear to use red or blue color coding. *The monkey groups are represented by colored monkey symbols, which are red and blue for the two IgG-infused groups.*
22. 622-624. This should probably go in the methods section. Were any validations done on a subset of samples to ensure that recently done assays provide similar numbers to those from historical assays?

*The lines recommended by the reviewer were moved to the Methods section. Both historical and contemporary assays were performed using the same protocol and included purified IgG from seropositive monkeys as a positive control that served as a control for variability between plates and assays run on different dates.*
23. Figure 6F. Please define abbreviations WVE, TCB. Why are there only two data points in the AF+ group? *Acronyms have been defined as recommended. We regret that the initial submission included an incorrect panel for the transfected cell binding result (the axis was set incorrectly and cut off several of the data points). This has been corrected. Thank you for such a thorough evaluation to notice this.*
24. Throughout – check that abbreviations are used consistently and only defined once (e.g., MKE, FBS, etc.). *Checked and corrected.*
25. Throughout – please define ED50, ID50, and ensure that these terms are applied appropriately. *Definitions added.*
26. Throughout – please provide sources or references and catalog numbers (where available) for all antibodies. *Catalog numbers or additional information on sourcing was added for all antibodies listed in the Methods section.*

Reviewer #3:

Typo line 44 “..the levels virus” *Corrected*
Suggest to revise line 53 “vaccines that confer levels of pre-existing immunity”. Unclear what this description means. *This line has been revised to read “confer protective levels of immunity to CMV-naïve mothers that can prevent primary infection and/or congenital transmission“.*
Provide greater specificity line 77 “CMV evolution has been shown to track host evolution”. Unclear what this description means. *We agree with the reviewer that this statement was unclear. We altered the manuscript and our statement now reads: “Furthermore, the continuous immunological arms race between CMVs and their host species has resulted in an ongoing, mutual virus/host co-adaptation reflected in the congruence of NHP and NHP-CMV phylogenetic trees and strict species specificity.” We are trying to express the sentiment that while NHPs are the closest usable animal model to the human host, the same is true on the virus side where RhCMV is the closest relative to HCMV that can be used in an in vivo model.*

What is a “true peak”? Perhaps a clear or unambiguous peak is meant? *Revised. The intention was to convey that the responses are still on an upward trajectory through the time points collected.*
Line 154. What is “pre-existing, potent antibody infusion”? Potent antibody infusion in advance of the challenge? *Yes, this has been revised for clarity.*
Line 199. Tense change “are clustering”. *Corrected.*
Revise sentence beginning on line 233 for clarity. *Revised to “Yet, this finding does not diminish the potential impact of vaccine-elicited immunity prior to child-bearing years that could reduce the risk of maternal infection and/or congenital transmission.“*
Line 238. Sentence is a fragment. *Corrected.*
Line 286. “factors that may play influence”. Needs revision. *Revised to “factors that influence“.*
Line 290 – “…immunity to primary infection will likely not be protective..” Because of the possible interpretation of immunity to mean protection rather than “immune responses”, this sentence is confusing. Revision suggested. *Revised to “humoral responses”.*

Line 290 – Inaccurate. Scope of this claim needs to be narrowed. The clinical evidence shows that natural immune responses to primary infection during pregnancy are not sufficient. This study demonstrates something similar in an NHP model. *Revised to “These results from our NHP model suggest …“.*

Figure 6 – define WVE, PC, TCB acronyms. *The graph labels were changed to match the ones in Figure 5 and remaining acronyms defined in the figure legend.*

Overall, a solid grammatical proofreading seems in order. *This has been completed by a thorough review by multiple authors.*
