# Supplementary material for: Relationship of maternal cytomegalovirus-specific antibody responses and viral load to vertical transmission risk following primary maternal infection in a rhesus macaque model
Source: PLoS Pathog. 2023 Oct 23;19(10):e1011378. doi: 10.1371/journal.ppat.1011378 (PMC10621917; doi:10.1371/journal.ppat.1011378)
Supplement: Peer review history

# Peer review: Decision Letter 0

**DOI:** 10.1371/journal.ppat.1011378.r001

**Authors:** Blossom Damania, Eain A Murphy

22 May 2023

Dear Dr. Permar,

Thank you very much for submitting your manuscript "Relationship of maternal cytomegalovirus-specific antibody responses and viral load to vertical transmission risk following primary maternal infection in a rhesus macaque model" for consideration at PLOS Pathogens. As with all papers reviewed by the journal, your manuscript was reviewed by members of the editorial board and by several independent reviewers. The reviewers appreciated the attention to an important topic. Based on the reviews, we are likely to accept this manuscript for publication, providing that you modify the manuscript according to the review recommendations.

Dr. Permar,

I hope this finds you well, Sally. We finally got the reviews returned on your manuscript. I do apologize for the extended review time but reviewers are hard to acquire during the spring/summer months and I gave extra time to the reviewers as they were experts in this field. All reviewers (and myself for that matter) are very supportive of this manuscript as it highlights a very novel and important model system. There remain concerns that I believe can be addressed in an easy enough fashion and I have every confidence that providing clarity in a resubmission will result in a favorable decision. As such, the decision for this manuscript is Minor Modify. I look forward to receiving the revised version.

Cheers,

Eain Murphy

Please prepare and submit your revised manuscript within 30 days. If you anticipate any delay, please let us know the expected resubmission date by replying to this email.

When you are ready to resubmit, please upload the following:

[1] A letter containing a detailed list of your responses to all review comments, and a description of the changes you have made in the manuscript.

Please note while forming your response, if your article is accepted, you may have the opportunity to make the peer review history publicly available. The record will include editor decision letters (with reviews) and your responses to reviewer comments. If eligible, we will contact you to opt in or out

[2] Two versions of the revised manuscript: one with either highlights or tracked changes denoting where the text has been changed; the other a clean version (uploaded as the manuscript file).

Important additional instructions are given below your reviewer comments.

Thank you again for your submission to our journal. We hope that our editorial process has been constructive so far, and we welcome your feedback at any time. Please don't hesitate to contact us if you have any questions or comments.

Sincerely,

Eain A Murphy, Ph.D.

Academic Editor

PLOS Pathogens

Blossom Damania

Section Editor

PLOS Pathogens

Kasturi Haldar

Editor-in-Chief

PLOS Pathogens

​orcid.org/0000-0001-5065-158X

Michael Malim

Editor-in-Chief

PLOS Pathogens

orcid.org/0000-0002-7699-2064

***********************

Dr. Permar,

I hope this finds you well, Sally. We finally got the reviews returned on your manuscript. I do apologize for the extended review time but reviewers are hard to acquire during the spring/summer months and I gave extra time to the reviewers as they were experts in this field. All reviewers (and myself for that matter) are very supportive of this manuscript as it highlights a very novel and important model system. There remain concerns that I believe can be addressed in an easy enough fashion and I have every confidence that providing clarity in a resubmission will result in a favorable decision. As such, the decision for this manuscript is Minor Modify. I look forward to receiving the revised version.

Cheers,

Eain Murphy

Reviewer Comments (if any, and for reference):

Reviewer's Responses to Questions

**Part I - Summary**

Please use this section to discuss strengths/weaknesses of study, novelty/significance, general execution and scholarship.

Reviewer #1: This study by Otero et al. is highly significant as it provides the first in vivo model that appears to closely mimic human mother-to-fetus vertical transmission following a primary HCMV infection. Primary infection of pregnant persons results in an ~40% transmission rate. However, identifying immunological and/or virological factors that provide protection against vertical transmission following a primary HCMV infection is a major gap in the field largely due to the asymptomatic/mild nature of infection in healthy individuals and the lack of a biologically comparable in vivo model. The Permar group originally developed a non-human primate (NHP) model of vertical transmission by depleting maternal CD4 T cells prior of RhCMV challenge, which resulted in a 100% mother-to-fetus transmission rate. This current study adds to those initial findings by increasing the cohort size of fully immunocompetent seronegative rhesus dams to assess RhCMV transmission in healthy mothers. Remarkably, similar to HCMV vertical transmission rates, the investigators found an ~40% RhCMV transmission rate in seronegative rhesus dams, suggesting this model could identify key immunological and/or virological determinates of congenital CMV transmission in healthy pregnant persons. Using this model, the investigators perform standard virological and immunological assays to attempt to identify factors associated with promoting or opposing RhCMV vertical transmission. They found neither levels of maternal viremia nor humoral responses were associated with congenital CMV infection following a primary infection of healthy dams, which was unknown prior to development of this model. From a research standpoint, the individual experimental assays are straightforward and appropriate. However, the presentation of some of the data are difficult to follow at times, particularly the results combining both healthy and CD4-depleted dams. Nonetheless, this is a highly significant study describing a novel NHP vertical transmission model that will provide the means to address previously unanswerable questions about mechanisms/correlates of protection in healthy pregnancy persons following a primary CMV infection.

Reviewer #2: This manuscript describes use of the rhesus macaque model to determine possible humoral correlates of protection against congenital transmission of rhesus cytomegalovirus (RhCMV), an important model for human cytomegalovirus congenital disease. Twelve RhCMV-negative dams were infected IV with a cocktail of two RhCMV strains. Blood and amniotic fluid (AF) were collected weekly and analyzed for viral load. Plasma was assessed for RhCMV-specific antibodies, neutralizing antibodies, and antibodies mediating RhCMV-specific ADCP and ADCC. Five dams had PCR-positive signals for RhCMV DNA in AF while seven were negative out to 20 weeks post challenge. There were no apparent associations between maternal viral load or the above measured humoral responses with fetal transmission.

The authors then added to these studies data from 15 additional experimentally-infected RhCMV-negative dams from previously published studies that were in some cases CD4-depleted and infused prior to infection with two RhCMV hyperimmuneglobulin (HIG) preparations. In some cases data were incorporated from the previously published studies, while some assays were conducted contemporaneously using archival samples. Incorporation of these additional animals generated inconsistencies that could confound interpretation, particularly as animals were infected with different viral cocktails consisting of different strains and two different HIG infusion protocols were used.

The resulting “meta” analyses identified high maternal viral load and decreased/delayed development of gB- and PC-specific binding antibodies as associated with transmission, but these correlations were largely driven by the CD4-depleted group, which considerably confounded interpretation. However, the data overall suggested that antibodies can provide protection against fetal infection, but in the context of primary infection they may not be present early enough to prevent transmission.

In general the manuscript is well organized and clearly written. Materials and Methods are a bit sloppy; detailed suggestions for improvement are provided among the “specific comments”. There are a few broad topics, presented below, that the authors may wish to consider elaborating on in the Discussion.

There is evidence that in humans delayed maternal humoral responses after primary infection are associated with transmission (PMID: 28008685). There is also evidence that HIG can reduce transmission if given early, at high dose, and more frequently than standard regimens (PMID: 29947159). It is also surprising that the authors did not choose to measure antibody avidity, which has been hypothesized to facilitate virion transport across the placenta. In humans high avidity antibodies can take two to six months to evolve and timing can be variable. The rhesus studies spanned about three months and so it is possible that the late transmission events may be associated with lower antibody avidity.

Congenital transmission is a highly complex and dynamic process that evolves dramatically during the course of pregnancy. The rhesus studies described raise important questions regarding time of transmission relative to both infection and conception. The authors allude to fetal transmission occurring rapidly after IV challenge in the rhesus model, but their data do not seem to support this, as all but one of the AF-positive dams had their first AF-positive result four weeks or more after challenge. Can the fetus be infected early but virus not be detected in the AF for several more weeks? In humans is there evidence that transmission occurs shortly after initial primary maternal infection, or is it a more protracted process?

It is also important to consider that the mechanisms of transplacental transmission, and consequently immune protection, may be different in first vs third trimester. In humans CMV transmission to the fetus is more efficient in third trimester than in first, perhaps because the integrity of the placenta as a maternal-fetal blood barrier begins to decline late in pregnancy. Thus transmissions that occur late in pregnancy may simply be due to mechanical leakage, whereas those early in pregnancy may require more active mechanisms such as progressive spreading of virus infections across placental tissues or, as has been proposed, active transport of virions via low avidity non-neutralizing antibodies. In any case, and especially as third trimester transmissions are also associated with less severe disease, it may make sense to analyze data from early fetal infections separately from later infections. For example, 0-3 week AOC data could be correlated with fetal infections that occurred up to 28 days post infection, while 3+ week AOC data could be correlated with those that occurred late (70-84 days).

While cumulatively the results reported are largely negative and in some instances are insufficient to support robust conclusions, the importance of identifying correlates of immune protection against congenital CMV disease, the paucity of data from this valuable animal model, and the thoroughness of the humoral analyses presented render this work of high significance to the field.

Reviewer #3: Otero et al evaluate cytomegalovirus “transmission” in a limited number of rhesus macaques in order to define correlates of congenital CMV (cCMV) infection. cCMV is a highly significant disease in human populations, and insights into how risk of transmission might be reduced are a clear and compelling clinical need. To the extent possible by abstracting from an animal model, this study demonstrates that neither maternal plasma viremia nor humoral immune responses relate to detection of CMV in amniotic fluid. The study is small (12 animals) and relies on several simplifications (equating amniotic fluid viral detection with fetal transmission) and abstractions between both host (NHP vs humans) and pathogen (rhCMV vs hCMV).

Beyond grammatical tidying, the manuscript would be improved by a more serious discussion of the limitations of the study. Some suggests are listed below.

**********

**Part II – Major Issues: Key Experiments Required for Acceptance**

Please use this section to detail the key new experiments or modifications of existing experiments that should be absolutely required to validate study conclusions.

Generally, there should be no more than 3 such required experiments or major modifications for a "Major Revision" recommendation. If more than 3 experiments are necessary to validate the study conclusions, then you are encouraged to recommend "Reject".

Reviewer #1: 1. Figure 1C shows the timing of detection of vertical transmission in the amniotic fluid (AF). It would also be helpful to the reader to show RhCMV DNA copies and duration of viral load in the AF of the 5 dams that exhibited vertical transmission in order to get a full sense of the magnitude of transmission during pregnancy.

2. In figure 2, the area under the curve (AUC) presented for the full virion serology are difficult to interpret because of the use of AUC rather than EC50 or endpoint titers, especially since EC50 are used for the other panels. Can these data be presented as endpoint titers or EC50 to improve clarity. It’s unclear as to how the AUC results represent the absolute fold change in antibody levels (i.e., does a 10-fold change in AUC equal a 10-fold antibody abundance).

3. Maternal IgG transfer across the placenta provides protection for the fetus from infection. Reduced levels of maternal IgG in the fetal circulation could lead to vertical transmission. What are the levels of maternal IgG in the umbilical cord blood (fetal circulation)?

4. Given that one of the main functions of the placenta is to act as a barrier protecting the fetus from infection, placental pathology (gross and/or microscopic) should be provided comparing AF-positive and AF-negative dams.

5. In figure 6, the investigators combine data from a previous study using CD4 T cell depleted dams that were treat with or without passive RhCMV-specific IgG antibodies. However, combining data from immunocompetent dams with those from immunocompromised dams somewhat muddles the results. Plotting AUC data only from the immunocompetent dams separating those that exhibited vertical transmission versus those that did not would be far more informative. Immunocompromised dams that did or did not receive passive IgG infusions could be plotted in a separate figure.

Reviewer #2: None required, but the authors should consider looking at avidity.

Reviewer #3: Comment on the on the challenge? Differences between the viruses? Can they be read out independently?

Tabulate and comment on effect size in addition to statistical significance.

Comment on power given small study size. What magnitude of effect could have been reliably detected with 12 animals? Especially given the variance observed in viral loads and humoral responses?

Describe limitations of modeling CMV – different virus, different route and kinetics of infection, AF, differences in Ab biology between humans and rhesus, AF virus status as a proxy for fetal infection, etc.

**********

**Part III – Minor Issues: Editorial and Data Presentation Modifications**

Please use this section for editorial suggestions as well as relatively minor modifications of existing data that would enhance clarity.

Reviewer #1: 1. In figure 6, the investigators combine data from a previous study using CD4 T cell depleted dams that were treat with or without passive RhCMV-specific IgG antibodies. However, combining data from immunocompetent dams with those from immunocompromised dams somewhat muddles the results. Plotting AUC data only from the immunocompetent dams separating those that exhibited vertical transmission versus those that did not would be far more informative. Immunocompromised dams that did or did not receive passive IgG infusions could be plotted in a separate figure.

Reviewer #2: Specific comments:

1. 78-79. NHP CMVs include CMVs that infect Great Apes.

2. 83. “fraction of the time” is vague and also suggests a short time period rather than low incidence; suggest “rarely” or similar.

3. 141. Should be “Figure 3A, B”.

4. 144. “never reached”.

5. 148-149. Unclear what is meant by “dose-optimized”.

6. 152-154. It would be helpful to briefly describe the different virus strains used in these studies. Are there four different strains, or is FL a BAC clone of 180.92? Do they have relevant genetic features or cell tropisms (e.g., PC mutations)?

7. 164-176. This paragraph was very hard to follow. In particular, it is unclear what group of animals are referred to in lines 168-169, or what exactly is the “combined group”. Also, please clarify “higher magnitude” - compared to what group (presumably AF-negative, but it is not stated)?

8. 195. Do you mean Fig. 7 here?

9. 197. “suggested”.

10. 230-233. The importance of early HIG treatment is further supported by work from Klaus Hamprecht (PMID: 29947159).

11. 238-240. The AF data presented seem inconsistent with this statement as four of the five AF-positives were first detected >28 days post infection and two were not detected until >70 days. Are there other data to suggest that transmission in this model occurs “shortly following infection”?

12. 260-262. “high titer” compared to what?

13. 320-331. Please provide species, source or reference, and catalog numbers (where available) for all cell lines. Include 1RFs (line 346) in this section.

14. 335. Please clarify “reduced-FBS”.

15. 342-344. Please provide details or reference for virus storage and titration methods.

16. 346-348. Please provide references or brief details of BAC construction for FL-RhCMV, including strain.

17. 405-406. Please provide references or brief details about the plasmids used.

18. 425. Were these data background-corrected with negative plasma?

19. 445. Were these data also background-corrected with negative plasma?

20. 598-599. This statement is not accurate as one animal was infected only with 180.92 and 12 were infected with UCD52 and FL.

21. 608. This figure does not appear to use red or blue color coding.

22. 622-624. This should probably go in the methods section. Were any validations done on a subset of samples to ensure that recently done assays provide similar numbers to those from historical assays?

23. Figure 6F. Please define abbreviations WVE, TCB. Why are there only two data points in the AF+ group?

24. Throughout – check that abbreviations are used consistently and only defined once (e.g., MKE, FBS, etc.).

25. Throughout – please define ED50, ID50, and ensure that these terms are applied appropriately.

26. Throughout – please provide sources or references and catalog numbers (where available) for all antibodies.

Reviewer #3: Typo line 44 “..the levels virus”

Suggest to revise line 53 “vaccines that confer levels of pre-existing immunity”. Unclear what this description means.

Provide greater specificity line 77 “CMV evolution has been shown to track host evolution”. Unclear what this description means.

Tense change line 144. What is a “true peak”? Perhaps a clear or unambiguous peak is meant?

Line 154. What is “pre-existing, potent antibody infusion”? Potent antibody infusion in advance of the challenge?

Line 199. Tense change “are clustering”.

Revise sentence beginning on line 233 for clarity.

Line 238. Sentence is a fragment.

Line 286. “factors that may play influence”. Needs revision.

Line 290 – “…immunity to primary infection will likely not be protective..” Because of the possible interpretation of immunity to mean protection rather than “immune responses”, this sentence is confusing. Revision suggested.

Line 290 – Inaccurate. Scope of this claim needs to be narrowed. The clinical evidence shows that natural immune responses to primary infection during pregnancy are not sufficient. This study demonstrates something similar in an NHP model.

Figure 6 – define WVE, PC, TCB acronyms.

Overall, a solid grammatical proofreading seems in order.

**********

PLOS authors have the option to publish the peer review history of their article (what does this mean?). If published, this will include your full peer review and any attached files.

If you choose “no”, your identity will remain anonymous but your review may still be made public.

**Do you want your identity to be public for this peer review?** For information about this choice, including consent withdrawal, please see our Privacy Policy.

Reviewer #1: No

Reviewer #2: No

Reviewer #3: No

Figure Files:

While revising your submission, please upload your figure files to the Preflight Analysis and Conversion Engine (PACE) digital diagnostic tool, https://pacev2.apexcovantage.com. PACE helps ensure that figures meet PLOS requirements. To use PACE, you must first register as a user. Then, login and navigate to the UPLOAD tab, where you will find detailed instructions on how to use the tool. If you encounter any issues or have any questions when using PACE, please email us at figures@plos.org.

Data Requirements:

Please note that, as a condition of publication, PLOS' data policy requires that you make available all data used to draw the conclusions outlined in your manuscript. Data must be deposited in an appropriate repository, included within the body of the manuscript, or uploaded as supporting information. This includes all numerical values that were used to generate graphs, histograms etc.. For an example see here: http://www.plosbiology.org/article/info%3Adoi%2F10.1371%2Fjournal.pbio.1001908#s5.

Reproducibility:

To enhance the reproducibility of your results, we recommend that you deposit your laboratory protocols in protocols.io, where a protocol can be assigned its own identifier (DOI) such that it can be cited independently in the future. Additionally, PLOS ONE offers an option to publish peer-reviewed clinical study protocols. Read more information on sharing protocols at https://plos.org/protocols?utm_medium=editorial-email&utm_source=authorletters&utm_campaign=protocols

References:

Please review your reference list to ensure that it is complete and correct. If you have cited papers that have been retracted, please include the rationale for doing so in the manuscript text, or remove these references and replace them with relevant current references. Any changes to the reference list should be mentioned in the rebuttal letter that accompanies your revised manuscript. If you need to cite a retracted article, indicate the article’s retracted status in the References list and also include a citation and full reference for the retraction notice.

# Author comment: Author response to Decision Letter 0

**DOI:** 10.1371/journal.ppat.1011378.r002

25 Sep 2023

Attachment Submitted filename: 20220913 response to reviewers_v3.docx Click here for additional data file.

# Editor report: Decision Letter 1

**DOI:** 10.1371/journal.ppat.1011378.r003

**Authors:** Blossom Damania, Eain A Murphy

29 Sep 2023

Dear Dr. Permar,

We are pleased to inform you that your manuscript 'Relationship of maternal cytomegalovirus-specific antibody responses and viral load to vertical transmission risk following primary maternal infection in a rhesus macaque model' has been provisionally accepted for publication in PLOS Pathogens.

Before your manuscript can be formally accepted you will need to complete some formatting changes, which you will receive in a follow up email. A member of our team will be in touch with a set of requests.

Please note that your manuscript will not be scheduled for publication until you have made the required changes, so a swift response is appreciated.

IMPORTANT: The editorial review process is now complete. PLOS will only permit corrections to spelling, formatting or significant scientific errors from this point onwards. Requests for major changes, or any which affect the scientific understanding of your work, will cause delays to the publication date of your manuscript.

Should you, your institution's press office or the journal office choose to press release your paper, you will automatically be opted out of early publication. We ask that you notify us now if you or your institution is planning to press release the article. All press must be co-ordinated with PLOS.

Thank you again for supporting Open Access publishing; we are looking forward to publishing your work in PLOS Pathogens.

Best regards,

Eain A Murphy, Ph.D.

Academic Editor

PLOS Pathogens

Blossom Damania

Section Editor

PLOS Pathogens

Kasturi Haldar

Editor-in-Chief

PLOS Pathogens

​orcid.org/0000-0001-5065-158X

Michael Malim

Editor-in-Chief

PLOS Pathogens

orcid.org/0000-0002-7699-2064

***********************************************************

Dear Dr. Permar (Sallie),

Thank you for your recent resubmission of your manuscript to PLoS Pathogens. It is apparent that you have improved the offering (which in my own opinion was a very nice piece of work). I am happy to inform you that the manuscript will be Accepted for publication in PLoS Pathogens in this revised form. Again, congratulations on an important study.

Cheers,

Eain Murphy

Reviewer Comments (if any, and for reference):

# Editor report: Acceptance letter

**DOI:** 10.1371/journal.ppat.1011378.r004

**Authors:** Blossom Damania, Eain A Murphy

17 Oct 2023

Dear Dr. Permar,

We are delighted to inform you that your manuscript, "Relationship of maternal cytomegalovirus-specific antibody responses and viral load to vertical transmission risk following primary maternal infection in a rhesus macaque model," has been formally accepted for publication in PLOS Pathogens.

We have now passed your article onto the PLOS Production Department who will complete the rest of the pre-publication process. All authors will receive a confirmation email upon publication.

The corresponding author will soon be receiving a typeset proof for review, to ensure errors have not been introduced during production. Please review the PDF proof of your manuscript carefully, as this is the last chance to correct any scientific or type-setting errors. Please note that major changes, or those which affect the scientific understanding of the work, will likely cause delays to the publication date of your manuscript. Note: Proofs for Front Matter articles (Pearls, Reviews, Opinions, etc...) are generated on a different schedule and may not be made available as quickly.

Soon after your final files are uploaded, the early version of your manuscript, if you opted to have an early version of your article, will be published online. The date of the early version will be your article's publication date. The final article will be published to the same URL, and all versions of the paper will be accessible to readers.

Thank you again for supporting open-access publishing; we are looking forward to publishing your work in PLOS Pathogens. 

Best regards,

Kasturi Haldar

Editor-in-Chief

PLOS Pathogens

​orcid.org/0000-0001-5065-158X

Michael Malim

Editor-in-Chief

PLOS Pathogens

orcid.org/0000-0002-7699-2064
